# Does nest occupancy by birds influence the microbial composition?

**DOI:** 10.3389/fmicb.2023.1232208

**Published:** 2023-11-20

**Authors:** Jiajia Xin, Heqin Cao, Xiaoyang Bao, Canshi Hu

**Affiliations:** ^1^College of Life Sciences, Guizhou University, Guiyang, Guizhou, China; ^2^Forestry College, Guizhou University, Guiyang, Guizhou, China; ^3^Research Center for Biodiversity and Nature Conservation, Guizhou University, Guiyang, Guizhou, China

**Keywords:** nest microbiota, 16S rRNA, ITS, Japanese tit, artificial nest boxes

## Abstract

Nest microbiota plays a vital role in the breeding and development of birds, which not only provides protection to bird hosts but also negatively affects the host. At present, it is unclear whether the composition of the microbes in the nests is affected by nesting. For this reason, we hung artificial nest boxes to simulate the natural nesting environment and combined 16S rRNA and ITS high-throughput sequencing technology to further study the differences in microbial composition and richness between used nests and control nests of Japanese tits (*Parus minor*). The study found that the bacteria in used nests and control nests showed significant differences at the phylum level (*p* < 0.05). It is also worth noting that the predominant bacteria in used nests were Proteobacteria (51.37%), Actinobacteria (29.72%), Bacteroidetes (6.59%), and Firmicutes (3.82%), while the predominant bacteria in control nests were Proteobacteria (93.70%), Bacteroidetes (2.33%), and Acidobacteria (2.06%). Both used nests and control nests showed similar fungi at the phylum level, which consisted mainly of Ascomycota and Basidiomycota, although significant differences were found in their relative abundance between both groups. The results of alpha diversity analysis showed significant differences in bacteria between the two groups and not in fungi. However, the beta diversity analysis showed significant differences between both bacteria and fungi. In summary, our results showed that the used nests had a higher abundance of beneficial microbiota and a lower presence of pathogenic microbiota. Therefore, we speculate that birds will change the characteristics of the nest microbial composition in the process of nest breeding to ensure their smooth reproductive development.

## 1 Introduction

The relationship between birds and microbiota is a complex symbiotic association that encompasses various beneficial functions, such as nutrition, defense, and protection, for the host. However, it can also have detrimental effects on the host’s health (Costanzo et al., 2022). The gut microbiome plays a crucial role in the host animal (Bodawatta et al., 2021; Diez-Méndez et al., 2023), contributing to digestion and interacting with the immune system during the establishment and development of the microbiome (Borda-Molina et al., 2018; Broom and Kogut, 2018). Furthermore, the presence of parasitic microbiota on the skin can serve as a tangible impediment to the organism’s exposure to various external substances (Sanford and Gallo, 2013). An abundance of pathogenic bacteria in the atmosphere can lead to ailments such as respiratory inflammation and impairment of pulmonary function (Hanni et al., 2003). Additionally, numerous bacteria residing on avian plumage can deteriorate the pigmented feathers through the regulation of their microflora (Shawkey et al., 2005). In the case of nesting creatures, the microbiota inhabiting in the nest also exert an influence on the wellbeing of the host. It has been shown that animals are able to shape and sustain their nest microbial symbionts (Lucas et al., 2017) and that the nest bacteria can purify the microenvironment by producing antibacterial chemicals (Poulsen et al., 2011; Madden et al., 2013). Consequently, the nest microbial environment is closely related to the growth and health of animals.

Nests have stable microclimate conditions that provide suitable growth conditions for microbiota (Costanzo et al., 2022). Nest microbiota can interact with their hosts in many different ways and may undergo mutual transfer between birds and nest materials, which plays an important role in the growth and reproduction of birds (Pugh, 1996). Some bacterial groups may have beneficial effects in hosts by promoting their growth and development. For instance, several Actinobacteria have antibacterial activity and thus produce antibiotics that inhibit the invasion of a variety of potential pathogens (Photita et al., 2004; De Souza et al., 2013). Other bacterial groups are potentially pathogenic bacteria that can remain dormant in nests and feces for months. Breeding birds are at an increased risk of bacterial infections (Sonia González-Braojos et al., 2012). For example, *Aspergillus fumigatus*, isolated from nests, cause fungal infections in birds’ lungs and air sacs, and birds are particularly susceptible to these infections (Barathidasan et al., 2013). The factors affecting the microbial colonization in the nest are not clear; nest structure, nesting materials, and the adult bird’s growth may affect the richness of the microbial communities (Kornillowicz and Kitowski, 2018; Costanzo et al., 2022), and ectoparasites may also affect the bacterial environment of bird nests (Tomás et al., 2018). Nest microbiota may originate from the birds themselves, such as *Bacillus* on feathers and *Enterobacter cloacae* in feces (Aguirre et al., 1992; Riffel and Brandelli, 2006). Additionally, nest material may also be one of the main sources of microbes in the nest during bird reproduction (Goodenough and Stallwood, 2010). Therefore, the nest microbiota has a significant effect on the growth, health, reproduction, and development of birds, but the current research based on the nest microorganisms is not extensive enough.

To protect the normal development of the offspring, the adults usually modify the internal environment of the nest during the breeding process of the birds. Costanzo et al. (2022) found that the microbiota in the nest may affect the gut microbes of the lesser kestrel (*Falco naumanni*). A variety of potentially pathogenic microbiota were also isolated in the nests of alpine vulture (*Gyps himalayensis*), warbler (*Troglodytes aedon*), and some wetland birds, which may affect chick development (David et al., 1998; Barathidasan et al., 2013; Korniłłowicz and Kitowski, 2013). In addition, the bacterial communities on the eggshell may also influence the hatching success and the status of the chicks (Peralta-Sánchez et al., 2012; Song et al., 2023). Studies have found that the use of green plants and feathers as nesting materials may be a self-medication strategy because microbes on feathers produce antimicrobials and different color feathers have different effects on hatching success (Peralta-Sánchez et al., 2012, 2014; Ruiz-Castellano et al., 2019). Green plants can produce some volatile compounds that provide favorable conditions to protect the offspring from pathogenic infections (Ruiz-Castellano et al., 2016). In different reproductive stages, adult birds are also selective to nest materials. For example, starlings (*Sturnus unicolor*) preferentially choose feathers in the early spawning stage, while aromatic plants are preferentially chosen as nesting sites (Ruiz-Castellano et al., 2017).

The interaction between microbial communities and birds play a central role in the evolution of their life-history traits (Soler et al., 2015). To date, the relationship between birds and microbiota has focused on gut microbes, and the microecology within the nest has not been extensively studied. In this study, we aim to investigate whether birds, while occupying the nests, can cause microbial changes. We hypothesized that, in the relatively stable nests, birds will modify the microecological environment in the nest during their nesting period to ensure the success of reproduction. Therefore, we could anticipate a higher abundance of beneficial microbiota and a lower presence of potentially pathogenic microbiota in occupied nests. Understanding the changes in the microbiota of bird nests will contribute to bird diversity conservation and will increase our knowledge on the reproduction, development, and environmental adaptation of birds. Additionally, it can help to elucidate the interplay between birds and their microbiota.

## 2 Materials and methods

### 2.1 Study area

During the bird-breeding period from April to July 2021, Japanese tits were attracted by hanging artificial nest boxes in the secondary forest of Guizhou University’s campus (106°39′29.20″N 26°26′34.85″E, at 1108–1144 m altitude, in Guiyang, Guizhou Province, China). According to the size and breeding habits of Japanese tits, the dimensions of the nest boxes were 13 cm × 12 cm × 27 cm, the panel thickness was 1.5 cm, and the nest opening diameter was 4.0 cm. To reduce the influence of other disturbance factors, all nest boxes were placed in an environmentally homogeneous, continuous, and small forestland with minimal human disturbance. All nest boxes were hung randomly in the study area, and the distance between the nest boxes was more than 50 m. During the monitoring period, the nest boxes were checked once a week in the early breeding stage and once every 2 days after the nest material appeared. Japanese tits are widely distributed in China and are typical secondary nest birds that are unable to dig their own tree hole. The breeding period of Japanese tits is from April to August each year, and the nesting period is generally 5–7 days. The nest is cup-shaped and the outer walls are mainly composed of moss.

### 2.2 Microbial sample collection

All the nest boxes were suspended in the secondary forest 3 months before the breeding period to ensure consistent initial status within the nest boxes. During the monitoring process of the breeding period, it was found that there were complete nest materials in nests, and in the subsequent monitoring, the Japanese tit exhibited spawning, hatching, brooding, and other behaviors, and such a nest was defined as used nests. Microbial samples of used nests were collected after the laying of the first eggs of Japanese tits (*N* = 9). During the same period, we defined the nest boxes without any material as control nests and collected samples of the microbes present (*N* = 9). We collected microbial samples using sterile rayon-tipped swabs (after being pre-moistened with sterile water) to wipe the edges of the nest boxes for 10 s and then crossed through the bottom in a cross pattern (Goodenough and Stallwood, 2010). Finally, all samples were placed in sterile sampling tubes and transferred to a cryogenic freezer set at −80°C.

### 2.3 DNA extraction and PCR amplification

According to the manufacturer’s instructions, total microbial DNA samples were extracted using the OMEGA Soil DNA Kit (M5636-02) (Omega Bio-Tek, Norcross, GA, United States). The DNA sample quality was assessed by agarose gel electrophoresis, and the concentration and purity of the DNA samples were determined using NanoDrop NC2000 spectrophotometer (Thermo Fisher Scientific, Waltham, MA, USA). PCR amplification of the bacterial 16S rRNA genes V3-V4 region was performed using the forward primer 338F (5’-ACTCCTACGGGAGGCAGCA-3′) and the reverse primer 806R (5’-GGACTACHVGGGTWTCTAAT-3′). The internal transcribed spacer (ITS) regions were amplified using the forward primer ITS5 (5’-GGAAGTAAAAGTCGTAACAAGG-3′) and the reverse primer ITS2 (5’-GCTGCGTTCTTCATCGATGC-3′). The reaction system of PCR contained 5 μL of buffer (5×), 0.25 μL of Fast Pfu DNA Polymerase (5 U/μl), 2 μL (2.5 mM) of dNTPs, 1 μL (10 uM) of each forward and reverse primer, 1 μL of DNA template, and 14.75 μL of ddH_2_O. Thermal cycling consisted of initial denaturation at 98°C for 5 min, followed by 25 cycles consisting of denaturation at 98°C for 30 s, annealing at 53°C for 30 s, and extension at 72°C for 45 s, with a final extension of 5 min at 72°C. The resulting PCR products were extracted from a 2% agarose gel, further purified using Vazyme VAHTSTM DNA Clean Beads (Vazyme, Nanjing, China), and quantified using the Quant-iT PicoGreen dsDNA Assay Kit (Invitrogen, Carlsbad, CA, United States). Purified amplicons were pooled in equal amounts, and pair-end 2 × 250 bp sequencing was performed using the Illumina platform with NovaSeq 6,000 SP Reagent Kit (500 cycles) at Shanghai Personal Biotechnology Co., Ltd. (Shanghai, China).

### 2.4 Bioinformatics and statistical analysis

The data obtained from Illumina NovaSeq sequencing underwent a rigorous process that involved quality filtering, denoising, merging, and removal of chimeras using the DADA2 plugin (Callahan et al., 2016). The sequences obtained above were merged based on 100% sequence similarity, and the characteristic sequence ASV was clustered along with the abundance data tables. To generate a Venn plot based on UPARSE, we calculated the number of OTU (operational taxonomic units) shared by each nest box sample. Additionally, we drew a sparse curve to reflect the rationality of the sample sequencing data. Then, species taxonomic annotation of bacteria was performed according to the SILVA database (Release 138, https://www.arb-silva.de/documentation/release138/), and the species taxonomic annotation of fungi was performed according to the UNITE database (Release 8.0, https://unite.ut.ee/). The alpha diversity index was calculated using mothur software, including the Pielou’s evenness (reflecting species evenness), Chao1 and observed species (reflecting species richness), and Shannon and Simpson (reflecting species diversity) indices. The analysis of similarities (ANOSIM) was used to test whether the grouping was statistically significant. The beta diversity analysis was performed using the unweighted UniFrac and Jaccard distance metrics to investigate changes in the structure of the microbial community between bacterial and fungal samples, and then, the principal coordinate analysis (PCoA) and hierarchical clustering analysis were performed to visualize these changes. Linear discriminant analysis effect size (LEfSe) was performed to detect differentially abundant taxa across groups. Correlations between microbial samples were visualized by calculating the Spearman rank correlation coefficients.

## 3 Results

### 3.1 Analysis of gene sequencing and OTUs

**Figure 1:**
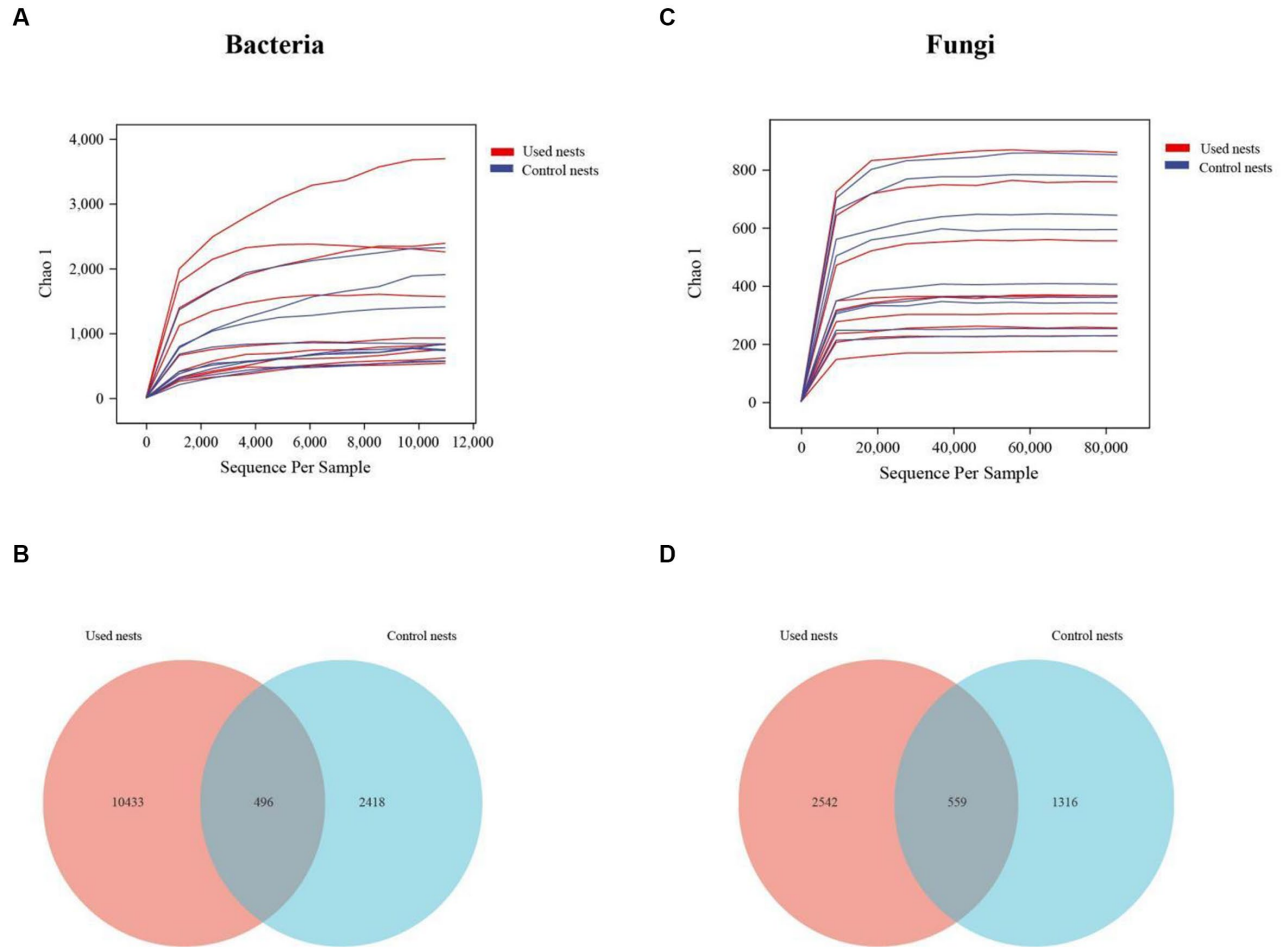
The rarefaction curves and Venn diagram in used nests and control nests of the Japanese tit. The rarefaction curves reflect the influence of sequencing depth on the diversity of observed samples. The curves began to plateau, indicating that the sequencing results were sufficient to reflect the diversity in the current samples. The Venn diagram shows the number of OTUs that are either shared or not shared between the used nests and control nests (based on 97% sequence similarity). **(A)** The rarefaction curves of bacteria. **(B)** The number of OTUs shared by bacteria. **(C)** The rarefaction curves of fungi. **(D)** The number of OTUs shared by fungi.

Based on 16S rDNA and ITS (internal transcribed spacer) gene sequencing, the nest microbiota composition of the Japanese tit was analyzed. After performing a series of processing steps with the sequencing results, a total of 2,263,238 valid sequences were obtained from 18 bacterial samples, ranging from 100,226 to 133,730 valid sequences per sample and the average sequence length of 361.20 bp (Supplementary Table S1). A total of 2,211,854 valid sequences were obtained from 18 fungal samples, ranging from 87,571 to 135,895 valid sequences per sample and the average sequence length of 235.94 bp (Supplementary Table S2). The rarefaction curves indicated that the sequencing data were large enough to effectively reflect most of the microbial diversity information in the sample (Figures 1A,C). The Venn diagram demonstrated that OTUs differed between the used nests and control nests. The total number of 496 and 559 OTUs were shared by bacteria and fungi between the used nests and control nests (Figures 1B,D).

### 3.2 Microbial composition of used nests and control nests

**Figure 2:**
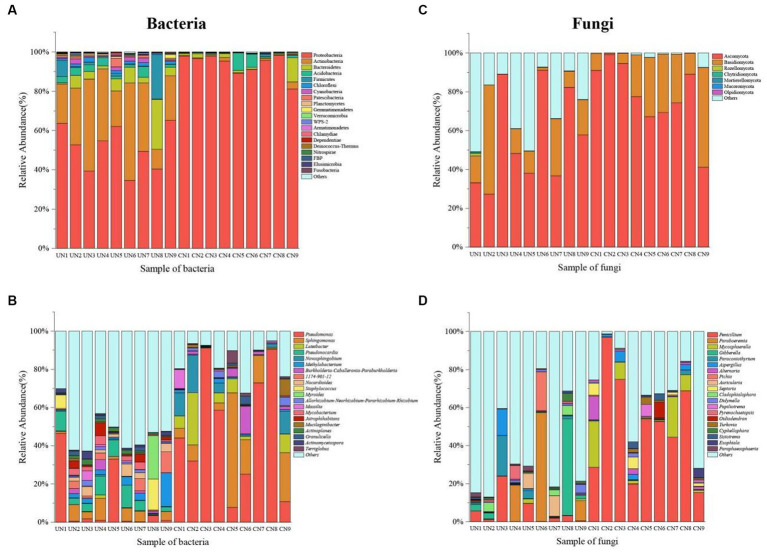
The histogram diagram shows the 20 most abundant taxa in used nests (UN 1–9) and control nests (CN 1–9). **(A)** Bacteria composition in all samples at the phylum level. **(B)** Bacteria composition in all samples at the genus level. **(C)** Fungi composition in all samples at the phylum level. **(D)** Fungi composition in all samples at the genus level.

**Figure 3:**
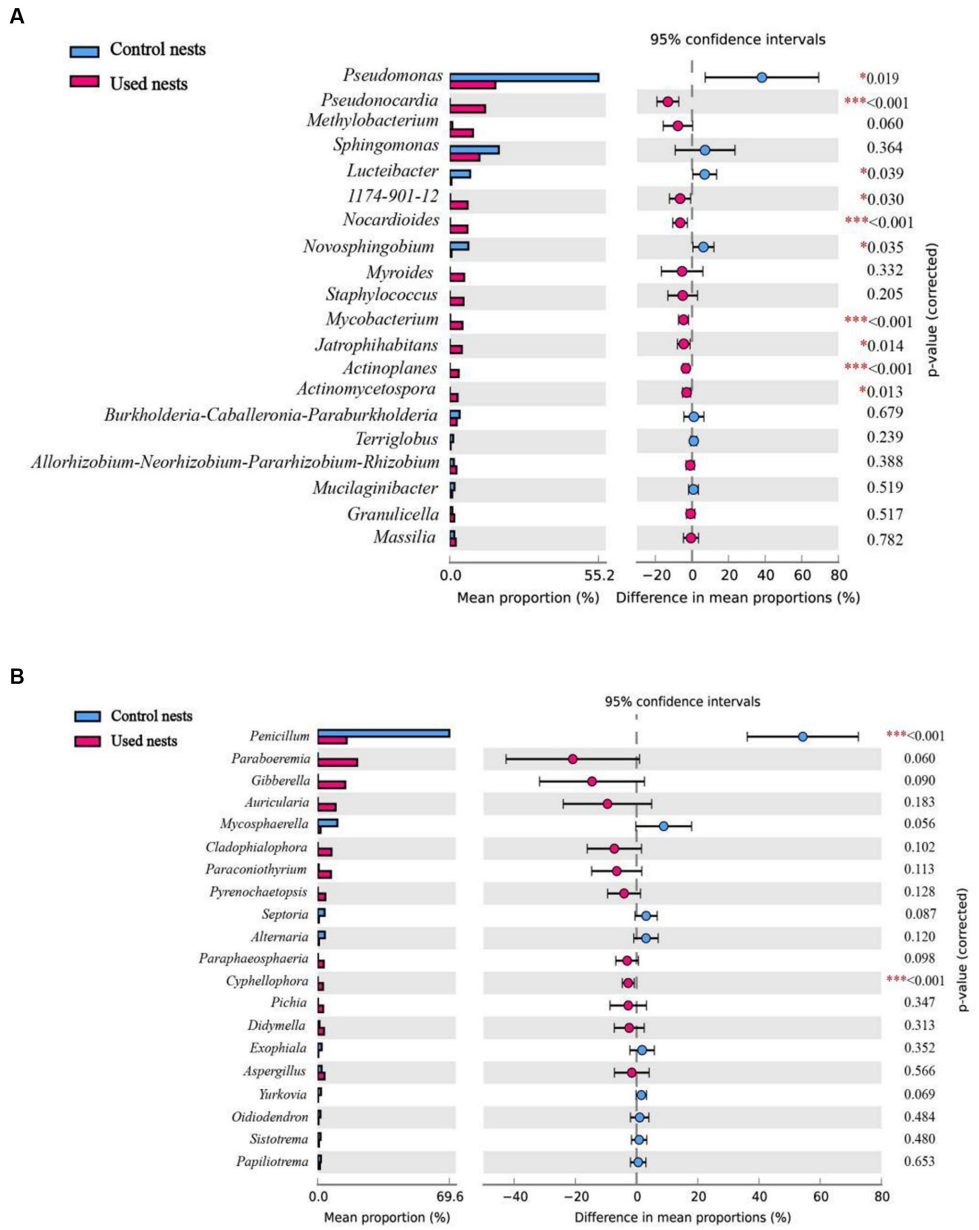
Differences in microbial composition at the genus level among the top 20 taxa of the used nests and control nests. Significant difference: *p < 0.05 was significant, **p < 0.01 was very significant, ***p < 0.001 was extremely significant. **(A)** Bacteria. **(B)** Fungi.

The taxonomic analysis revealed that a total of 27 bacterial phyla were identified in the microbiota 16S rRNA sequencing. Proteobacteria (51.37%), Actinobacteria (29.72%), Bacteroidetes (6.59%), Acidobacteria (2.77%), Firmicutes (3.82%), Chloroflexi (1.35%), Cyanobacteria (1.20%), and Patescibacteria (1.05%) were the dominant bacteria across all bacterial samples of used nests. In control nests, Proteobacteria (93.70%), Bacteroidetes (2.33%) and Acidobacteria (2.06%) were the dominant bacteria (Figure 2A; Supplementary Figure S1A). At the genus level, *Pseudomonas* (9.82%), *Pseudonocardia* (6.23%), *Sphingomonas* (4.85%), *Methylobacterium* (3.84%), *1,174–901-12* (2.93%), *Nocardioides* (2.75%), *Staphylococcus* (2.63%), *Myroides* (2.52%), *Mycobacterium* (2.03%), and *Jatrophihabitans* (2.02%) were the dominant bacteria across all bacterial samples of used nests. In control nests, *Pseudomonas* (48.13%), *Sphingomonas* (15.12%), *Luteibacter* (6.51%), *Novosphingobium* (5.79%), and *Burkholderia-Caballeronia-Paraburkholderia* (2.72%) were the dominant bacteria across all bacterial samples (Figure 2B; Supplementary Figure S1B). There were 10 genera that showed significant differences between used nests and control nests. Compared with control nests, the relative abundance of seven genera, including *Pseudonocardia*, *1,174–901-12*, *Nocardioides*, *Mycobacterium*, *Jatrophihabitans*, *Actinoplanes*, and *Actinomycetospora*, was significantly higher in used nests (*p* < 0.05). The relative abundance of *Pseudomonas*, *Lucteibacter*, and *Novosphingobium* was significantly lower in used nests than in control nests (Figure 3A).

The fungi of those samples were classified into seven phyla in ITS sequencing (Figure 2C; Supplementary Figure S2A). Both Ascomycota and Basidiomycota were the main components of the used nests and control nests, but they showed relative differences in abundance. The relative abundance of used nests was Ascomycota (55.96%) and Basidiomycota (16.84%). The relative abundance of control nests was Ascomycota (78.16%) and Basidiomycota (20.41%). At the genus level, the dominant fungi of the used nests were *Paraboeremia* (9.74%), *Gibberella* (6.70%), *Penicillium* (5.15%), *Paraconiothyrium* (3.20%), *Pichia* (2.27%), *Auricularia* (2.06%), *Aspergillus* (1.79%), and *Cladophialophora* (1.54%), whereas the dominant fungi of the control nests were *Penicillium* (50.68%), *Mycosphaerella* (7.45%), *Alternaria* (2.25%), *Septoria* (1.90%), and *Aspergillus* (1.46%) (Figure 2D; Supplementary Figure S2B). There were two genera that showed significant differences between used nests and control nests (*p* < 0.05). Specifically, *Cyphellophaeria* was found to be significantly higher in used nests, while *Penicillium* was significantly lower in used nests (Figure 3B).

### 3.3 Analysis of diversity between used nests and control nests

To compare evenness, richness, and diversity between used nests and control nests, we calculated the alpha diversity index (Pielou’s evenness, Chao1, observed species, Shannon, and Simpson indices). The alpha diversity results showed that the Pielou’s, Chao1, observed species, Shannon, and Simpson indices of the bacterial sample in used nests were significantly higher than those in control nests (*p* < 0.01) (Table 1). There were no significant differences in the alpha diversity indices of the fungal samples (*p* > 0.05) (Table 1).

**Table 1:** Alpha diversity indices of used nests and control nests.

Alpha diversity	Bacteria			Fungi		
	Used nests	Control nests	*p*-value	Used nests	Control nests	*p*-value
Pielou’s evenness	0.80 ± 0.11	0.48 ± 0.19	*p* = 0.00^***^	0.56 ± 0.10	0.48 ± 0.16	*p* = 0.23
Chao1	1923.33 ± 881.36	686.27 ± 115.28	*p* = 0.00^***^	564.12 ± 256.02	360.21 ± 154.87	*p* = 0.06
Observed species	1527.17 ± 614.91	468.20 ± 90.98	*p* = 0.00^***^	557.89 ± 251.40	356.30 ± 153.40	*p* = 0.06
Shannon	8.41 ± 1.54	4.28 ± 1.75	*p* = 0.00^***^	5.08 ± 1.09	4.07 ± 1.53	*p* = 0.15
Simpson	0.96 ± 0.06	0.74 ± 0.28	*p* = 0.01^***^	0.86 ± 0.10	0.76 ± 0.25	*p* = 0.40

### 3.4 The difference analysis between used nests and control nests

The analysis of similarities (ANOSIM) showed differences in nest microbiota between used nests and control nests (bacteria: R = 0.759, *p* = 0.001; fungi: R = 0.693, *p* = 0.001; Supplementary Figures S3A,B). The results revealed that the intergroup differences were greater than the intragroup differences between used nests and control nests and that there were significant differences in the microbial composition between the two groups (*p* < 0.05).

**Figure 4:**
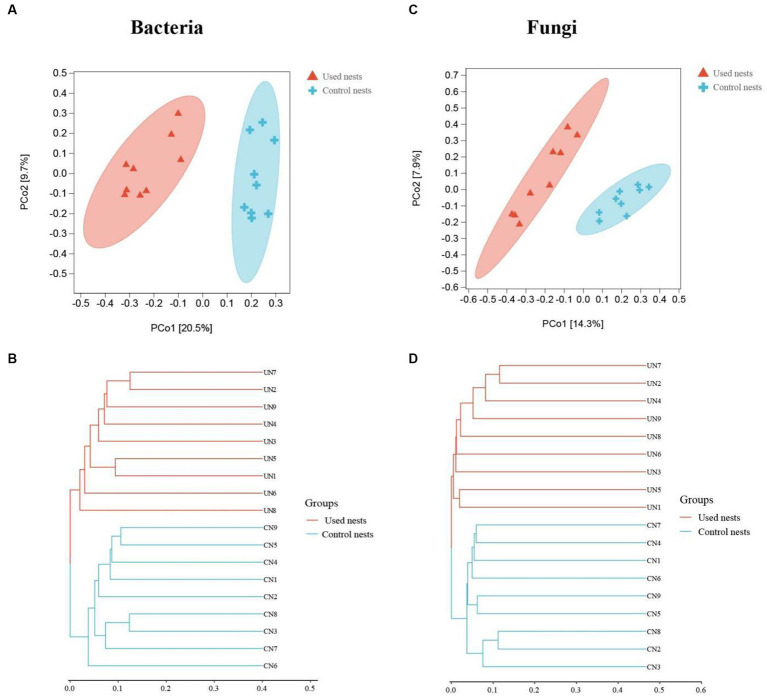
Plots of the principal coordinate analysis (PCoA) of bacteria **(A)** and fungi **(C)**. Samples in the same group are represented by the same color and shape. The x-axis and y-axis represent the first and second primary coordinates, respectively. The percentages in square brackets of the axis represent the proportion of the sample distance matrix that the corresponding axis can interpret. The distance between sample points indicates the similarity of microbial communities in the samples, and the closer the sample points are to each other, the more similar the two samples are. The hierarchical clustering analysis of bacteria **(B)** and fungi **(D)**. The panel is a hierarchical clustering tree diagram, in which samples are clustered according to their similarity, and the shorter the branch length between samples, the more similar the two samples are. Used nests: UN 1–9; Control nests: CN 1–9.

The beta diversity analysis was performed to investigate the structural variation of microbial communities. The PCoA and hierarchical clustering analyzes showed that there were significant differences between used nests and control nests (Figure 4). The PCo1 and PCo2 explained 20.5 and 9.7% (bacteria) and 14.3 and 7.9% (fungi) of the differential contribution rate, respectively (Figures 4A,C). As illustrated in Figures 4B,D, the same group showed obvious intragroup aggregation, while the used nests and control nests samples showed obvious separation, which means that the microbial communities in used nests and control nests samples have a great number of differences.

**Figure 5:**
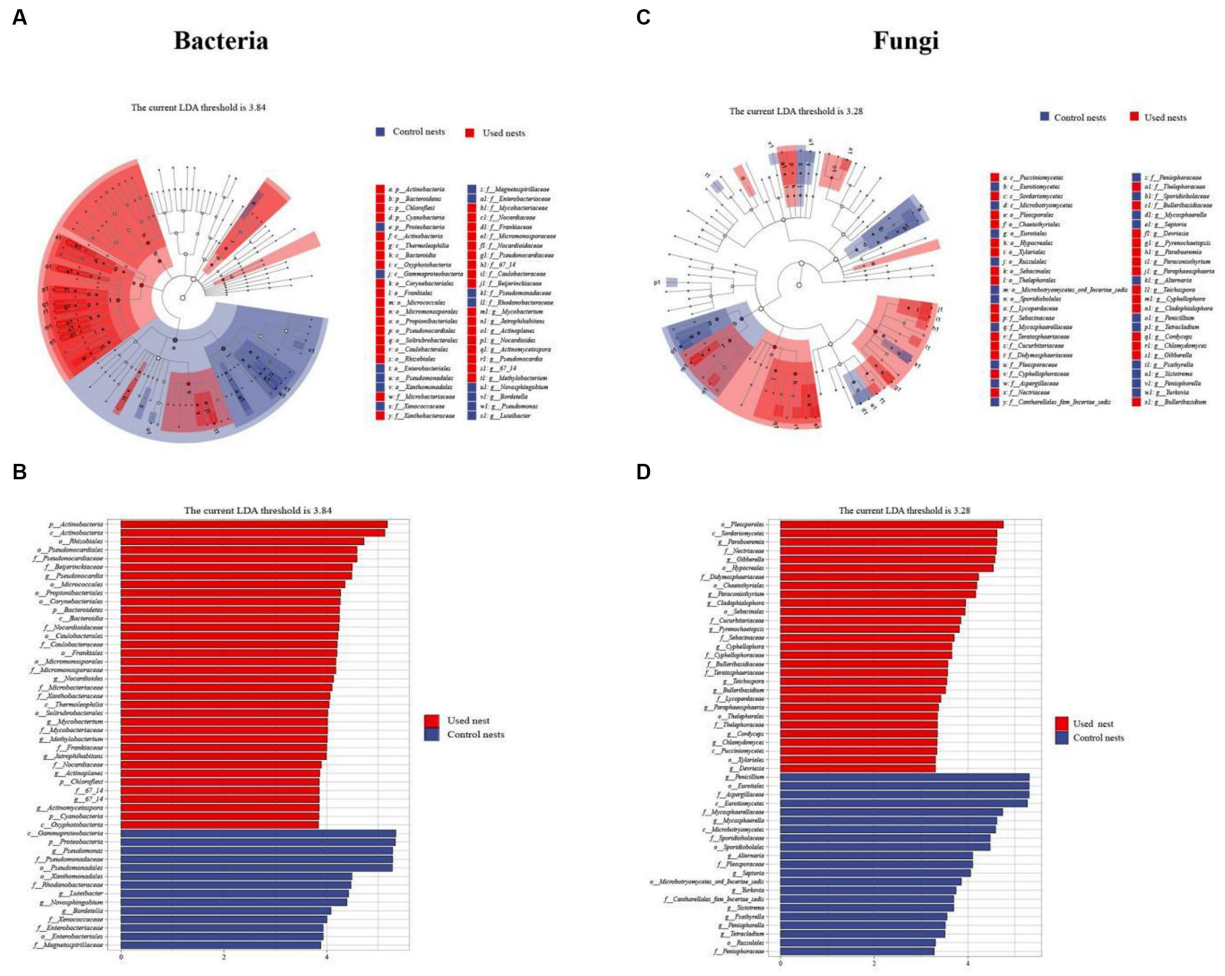
LEfSe analysis. **(A,C)** The cladograms showed the taxonomic hierarchical relationship ranging from phylum to genus level major taxa between used nests (red) and control nests (blue). **(B,D)** The plots show microbiota with significant differences between used nests (red) and control nests (blue).

To identify the biomarkers with statistical differences of nest microbiota in different groups, we performed the LEfSe analysis between used nests and control nests (Figure 5). The results of the line discriminant analysis (LDA) showed 50 significant biomarkers related to bacteria. Out of these, 36 biomarkers were found in used nests (LDA > 3.84), distributed among Actinobacteria (25), Proteobacteria (6), Cyanobacteria (3), and Bacteroidetes (2). Control nests showed 14 bacterial biomarkers (LDA > 3.84) distributed in Proteobacteria (13) and Cyanobacteria (1) (Figure 5B). The results related to fungi revealed that there were 29 biomarkers of used nests (LDA > 3.28) and 21 biomarkers of control nests (LDA > 3.28), and the major microbiota were Ascomycota (21 in used nests and 10 in control nests) and Basidiomycota (8 in used nests and 11 in control nests) (Figure 5D). Then, the diagrams of taxonomic clade were produced to identify major microflora. As shown in the cladogram, biomarkers of different classification levels were significantly different between used nests and control nests (Figures 5A,C).

### 3.5 Correlation analysis of bacteria and fungi

**Figure 6:**
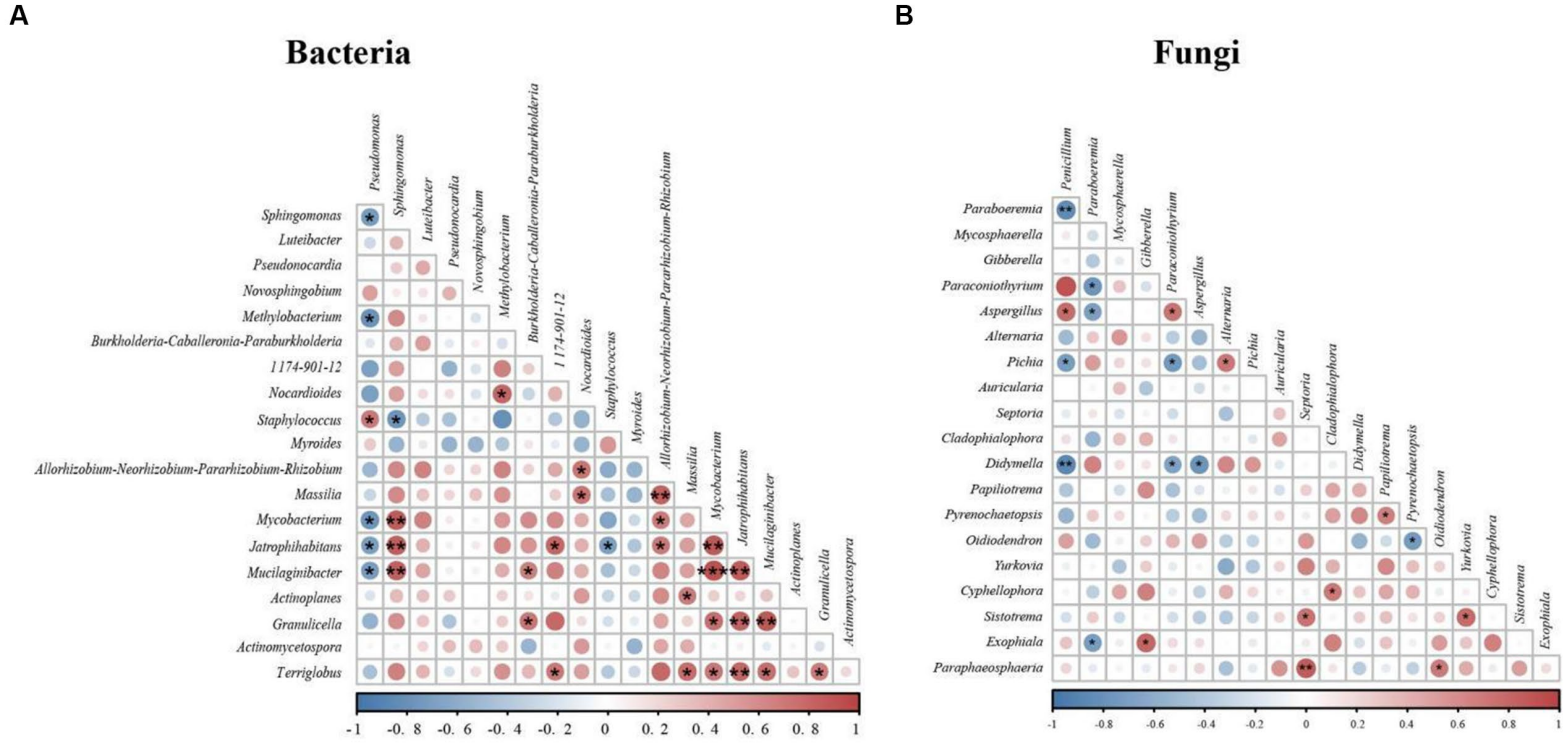
Correlation heatmap of used nests: red indicates a positively correlation, and blue indicates a negative correlation. **(A)** Correlation heatmap of bacteria. **(B)** Correlation heatmap of fungi.

To understand the species correlation between nest microbiota, we selected the top 20 bacterial and fungal genera of used nests in total horizontal abundance and calculated their Spearman rank correlation coefficients (Figure 6). The correlation heatmap showed 26 strong positive correlations and 7 strong negative correlations among bacterial genera (Figure 6A), while there were 10 strong positive correlations and 10 strong negative correlations among fungal genera (Figure 6B). These results indicated that there was significant correlation between microbiota in Japanese tit nests.

## 4 Discussion

The internal environment of the nests is relatively stable and suitable for a variety of microbiota (Costanzo et al., 2022). The species diversity and abundance of the nest microbiota play an important functional role in maintaining the normal physiology of the host. At the same time, the nest microbiota is also affected by the host. This study used 16S rRNA and ITS sequencing to compare the differences in the microbial composition between used nests and control nests and showed that the microbial diversity of the used nests was higher than that of the control nests, which could be related to the nesting of the birds.

### 4.1 Characteristics of the bacterial composition within the Japanese tit nests

Bacteria are regarded as an important driver of the host’s life history (Kevin et al., 2019), and they are closely related to the survival and development of birds. Our results showed that bacterial diversity of the used nests was significantly higher than that of the control nests (*p* < 0.01). At the phylum level, the main dominant bacteria in the used nests were Proteobacteria (51.37%), Actinobacteria (29.72%), Bacteroidetes (6.59%), and Firmicutes (3.82%), while the main dominant bacteria in the control nests were Proteobacteria (93.70%), Bacteroidetes (2.33%), and Acidobacteria (2.06%) (Figure 2A). This result is consistent with the previous studies of nest microbiota (Goodenough and Stallwood, 2010; Costanzo et al., 2022). Among these bacteria, some can form good symbiotic relationships with the host, for example, Actinobacteria, a common environmental microbiota with antimicrobial activity (Kielak et al., 2016) that produces antibiotics to inhibit the invasion of various potential pathogens (De Souza et al., 2013; Arango et al., 2016). Hooper (2004) has reported that Bacteroidetes can promote the development of the immune system, enhance the host immunity, and then make its internal environment to achieve balance. Firmicutes has many genes responsible for the fermentation of dietary fiber that contribute to homeostasis after the interaction with the intestinal mucosa (Sun et al., 2022). Compared with the control nests, the relative abundance of the beneficial bacteria such as Actinobacteria, Bacteroidetes, and Firmicutes in the used nests was higher. Therefore, we speculate that Japanese tit may increase the abundance of beneficial bacteria in the microecological environment of the nest to ensure the success of reproduction and the healthy growth of chicks. However, other bacteria, such as Proteobacteria (a common pathogenic bacteria), can have negative effects on the host, by causing diseases such as respiratory tract infection and inflammation, which affects the ecological balance of the intestinal microbial community (Shin et al., 2015; Guan et al., 2018). Our results showed that the relative abundance of Proteobacteria in used nests (51.37%) was lower compared with the control nests (93.70%). This suggests that, after the beginning of reproduction, the nest microecological environment may be changed, resulting in a decrease of pathogenic bacteria in the nest.

At the genus level, the bacterial composition of used nests and control nests also varied greatly. The main dominant genera of used nests were *Pseudomonas* (9.82%), *Pseudonocardia* (6.23%), *Sphingomonas* (4.85%), and *Methylobacterium* (3.84%). The main dominant genera in control nests were *Pseudomonas* (48.13%), *Sphingomonas* (15.12%), *Luteibacter* (6.51%), and *Novosphingobium* (5.79%) (Figure 2B). Previous studies have found that *Pseudonocardia* can not only form a good symbiotic relationship with plants and animals (Currie et al., 1999) but also can produce antibiotics and immunomodulatory agents (Dekker et al., 1998). The *Pseudonocardia* proportion of the used nests was significantly greater compared with the control nests (*p* < 0.001). This indicates that the bacterium may have a positive effect on the reproduction and development of Japanese tits. Then, we found that the main components of nests material were mosses and other plant fibers. Calatrava et al. (2018) had reported that *Methylobacterium* was a bacterium that promoted plant growth. Therefore, the ability of mosses to remain dry and intact in the nest box (Proctor et al., 2001) may be related to the biological characteristics of *Methylobacterium*. In addition, a small number of potential pathogenic bacteria existed in used nests, which may cause diseases in birds. For example, *Sphingomonas*, *Pseudomonas*, and *Staphylococcus* mostly existed in the environment and were all opportunistic pathogens causing infection (Silvanose et al., 2001; Yim et al., 2010; Fan et al., 2019). David et al. (1998) also isolated *Pseudomonas* and *Staphylococcus* in old house wren (*Troglodytes aedon*) nests and suggested that these genera may be closely related to the nesting environment of birds. Spearman correlation analysis showed that *Methylobacterium* had a significant negative correlation with *Pseudomonas* (*p* < 0.05), *Staphylococcus*, and *Sphingomonas* (Figure 6A). Therefore, the relative abundance of *Pseudomonas* in used nests was significantly lower than that in control nests, which might be due to the introduction of beneficial bacterium *Methylobacterium*, which inhibited the growth of *Pseudomonas*. The relative abundance of *Staphylococcus* in used nests was higher compared with control nests, which might be related to the introduction of *Sphingomonas* and other bacteria. In conclusion, the analysis of the bacterial composition of used and control nests found that the bacterial diversity in the used nests was significantly higher than that in the control nests (*p* < 0.05), with higher abundance of beneficial bacteria and a lower presence of potential pathogenic bacteria.

### 4.2 Characteristics of fungal composition within Japanese tit nests

Fungi are widely found in nature. They are not only a major component of many animals’ diet (Wallis et al., 2012) but also an important part of nest microbiota (Goodenough and Stallwood, 2010). At the phylum level, the composition of used nests and control nests was similar, and the dominant fungi were Ascomycota and Basidiomycota (Figure 2C). Ascomycota and Basidiomycota are the largest phyla in the fungal kingdom, with a wide range of biological activities that survive under a variety of conditions, some of which produce lethal toxins (Lin et al., 2019) and were the source of animal and human diseases (Lutzoni et al., 2001). Our results showed that the relative abundance of Ascomycota and Basidiomycota in the used nests was lower compared with the control nests. Overall, the growth of these fungi may be inhibited after the birds move into the nests.

At the genus level, there was a significant difference of the composition between used and control nests (Figure 2D). The main dominant genera in the used nests were *Paraboeremia* (9.74%), *Gibberella* (6.70%), *Penicillium* (5.15%), and *Paraconiothyrium* (3.20%). Among these fungi, *Paraboeremia* and *Gibberella* have been reported to be complex plant pathogens. Some strains can cause spots in plant leaves or stems (Jiang et al., 2017), whereas other strains can promote plant growth and development (Enrique et al., 1994). Therefore, *Paraboeremia* and *Gibberella*, which were only detected within the used nests, possibly related to the introduction of plant nest materials. In addition, Wang et al. (2021) reported that *Paraconiothyrium* was widely distributed, has multiple host habitats, and has potential application as biocontrol agents, bioreactors, and antibiotic producers. This fungus in used nests was significantly higher than that in control nests, indicating that it might be beneficial for the reproduction of birds. Compared with the used nests, *Penicillium* (50.68%) and *Mycosphaerella* (7.45%) accounted for higher relative abundance in control nests, which were all common pathogens and easily caused diseases in animals and plants (Costa and Oliveira, 1998; Arzanlou et al., 2008). Consequently, they might cause chick infection during reproduction and reduced reproductive success. The correlation analysis showed that there was a significant negative correlation between *Paraboeremia* and *Penicillium* in used nests (*p* < 0.01) (Figure 6B), which might inhibit the growth of some potential pathogenic fungi such as *Penicillium*. In conclusion, in terms of fungal composition characteristics, used nests had a higher abundance of beneficial fungi and a lower presence of potential pathogenic fungi.

### 4.3 Source of microbiota within the used nests

Overall, the composition of bacteria and fungi in the nests changed significantly after Japanese tits moved into the artificial nest boxes, and the overall findings was that there were more beneficial bacteria (*Pseudonocardia*, *Methylobacterium*, *Paraconiothyrium*, *Paraboeremia*, and *Gibberella*) and fewer potential pathogenic bacteria (*Pseudomonas*, *Sphingomonas Staphylococcus*, and *Penicillium*). The sources for this change were unclear, but it was noteworthy that the nesting material and the introduction of adult birds.

The increase in beneficial microbiota in used nests may be related to the introduction of mosses. Previous studies have shown that nests were susceptible to contamination by ectoparasites, fungi, viruses, or bacteria, and that birds brought plant fragments with specific chemical compositions to the nest to provide a healthier nest environment by repelling or killing the parasites in the nests (Clark and Mason, 1985). Since the first report of Campbell and Ferguson (1972) was published, many field surveys had found that birds tend to choose mosses as the main component of the nest material (Mennerat et al., 2009; Zabłotni et al., 2020). In our study, we found that the main component of Japanese tit nest material was also mosses. Previous studies have shown that mosses had antibacterial effects (Petit et al., 2002), which could reduce the risk of pathogenic infection in birds, thus providing benefits to the growth of chicks (Mennerat et al., 2009). In addition, the Spearman correlation analysis indicated that there were significant associations between many microbiota (Figure 6), so there might also be interactions between microbiota, which jointly affected the composition changes of microbiota in the nests. By introducing mosses and other plant nest materials, their symbiotic bacteria (*Paraboeremia*, *Gibberella*, and *Methylobacterium*) might inhibit the growth of some potential pathogenic bacteria (*Penicillium* and *Pseudomonas*). Therefore, we speculate that adult birds may have the ability to actively select moss as the nest material during the breeding process so as to modify the favorable microecological environment in the nest.

A small amount of potential pathogenic microbiota present in used nests may originate from air, the body surface, or the intestine of adult birds. *Staphylococcus* and *Pseudomonas* were frequently detected in airborne microorganisms (Fan et al., 2019). Additionally *Pseudomonas* was also found in the feathers of the eastern blue bird (*Sialia sialis*) and the feces of other wild birds (Brittingham et al., 1998; Shawkey et al., 2005). Furthermore, we detected a small amount of *Aspergillus* (1.79%) in used nests, a common avian pathogenic bacterium that caused fungal infection in the lungs and airbags (Barathidasan et al., 2013). *Aspergillus* had been found in the intestines and feces of birds such as Japanese quail (*Coturnix japonica*) (Huff et al., 1992), wild turkey (*Meleagris gallopavo*) (Quist et al., 2000), and house sparrow (*Passer domesticus*) (Lawson et al., 2006). Therefore, air, feathers, and feces are also important sources of nest microbes, and these sources are closely related to the lifecycle of birds. The potential pathogenic bacteria and fungi in the nest, although harmful to chicks, are more common in birds and have less relative abundance, which may have limited influence on the growth and survival rate of nestlings during the brooding period.

## 5 Conclusion

This study showed that there were significant differences in the microbial composition between used the nests and control nests of Japanese tits. The microbial diversity of used nests was significantly higher compared with the control nests. There were more beneficial microbiota and less pathogenic microbiota in used nests, and the proportion of pathogenic microbiota in control nests was relatively higher. This indicated that the Japanese tits have changed the species composition, diversity, and richness of the microbial community in the nests to ensure the smooth progress of reproductive development. Changes in the composition of microbiota within the nests may be influenced by nesting materials, and adult birds may have the ability to actively modify the microecological environment within the nest. At present, there are few studies on microbiota in nests. Therefore, this study is the first to analyze and compare the microbiota diversity of the used nests of Japanese tits and control nests. This study will contribute to a deeper understanding of the influence of birds on microbiota in nests and will provide a theoretical basis for understanding the response relationship between birds and microbiota.

## Data availability statement

The datasets presented in this study can be found in online repositories. The names of the repository/repositories and accession number(s) can be found in the article/Supplementary material.

## Ethics statement

The animal study was approved by Guizhou University Subcommittee of Experimental Animal Ethics. The study was conducted in accordance with the local legislation and institutional requirements.

## Author contributions

JX and CH contributed to the experimental design and conception of the study and wrote the paper. JX, HC, and XB performed the field sample collection and part of the data analysis. CH and HC reviewed the manuscript and provided comments. All authors contributed to the article and approved the submitted version.
