# Supplementary material for: Does nest occupancy by birds influence the microbial composition?
Source: Front Microbiol. 2023 Nov 20;14:1232208. doi: 10.3389/fmicb.2023.1232208 (PMC10694247; doi:10.3389/fmicb.2023.1232208)
Supplement: Supplementary file 1 [file Data_Sheet_1.docx]

Supplementary Material

**Whether nest occupancy by birds influences microbial composition？**

**Jiajia Xin^1^, Heqin Cao^2^, Xiaoyang Bao^1^, Canshi Hu^1*^**

*** Correspondence:** Canshi Hu: cshu@gzu.edu.cn

# Supplementary Figures and Tables

## Supplementary Tables

**Supplementary Table 1 Statistical table of sequencing volume per bacterial sample**

| \| Sample \| Input \| Filtered \| Denoised \| Merged \| Non-chimeric \| Non-singleton \| \| --- \| --- \| --- \| --- \| --- \| --- \| --- \| \| UN1 \| 135396 \| 126250 \| 124094 \| 118141 \| 109635 \| 108908 \| \| UN2 \| 141828 \| 134865 \| 131026 \| 123478 \| 116729 \| 115550 \| \| UN3 \| 141235 \| 133022 \| 127085 \| 112293 \| 93342 \| 90434 \| \| UN4 \| 137124 \| 129746 \| 125737 \| 117037 \| 109357 \| 108342 \| \| UN5 \| 135938 \| 127507 \| 125699 \| 116480 \| 111281 \| 110988 \| \| UN6 \| 133221 \| 123670 \| 117083 \| 107622 \| 100260 \| 98011 \| \| UN7 \| 141074 \| 134268 \| 130428 \| 122130 \| 114190 \| 113067 \| \| UN8 \| 136390 \| 129551 \| 126123 \| 120632 \| 78452 \| 76224 \| \| UN9 \| 135808 \| 129382 \| 127518 \| 124773 \| 121410 \| 120986 \| \| CN1 \| 141473 \| 133834 \| 132639 \| 131844 \| 118100 \| 117927 \| \| CN2 \| 140078 \| 129448 \| 128276 \| 122378 \| 88001 \| 87488 \| \| CN3 \| 138972 \| 127659 \| 126369 \| 116831 \| 114883 \| 114737 \| \| CN4 \| 133542 \| 125844 \| 124254 \| 122079 \| 97914 \| 97456 \| \| CN5 \| 143494 \| 135168 \| 133730 \| 131468 \| 94039 \| 93795 \| \| CN6 \| 127553 \| 120749 \| 119212 \| 113353 \| 93959 \| 93607 \| \| CN7 \| 146817 \| 134862 \| 133723 \| 124938 \| 114794 \| 114555 \| \| CN8 \| 140869 \| 131229 \| 130016 \| 122120 \| 115488 \| 115287 \| \| CN9 \| 107971 \| 101501 \| 100226 \| 95193 \| 83599 \| 83296 \| |
| --- | --- | --- | --- | --- | --- | --- | --- | --- | --- | --- | --- | --- | --- | --- | --- | --- | --- | --- | --- | --- | --- | --- | --- | --- | --- | --- | --- | --- | --- | --- | --- | --- | --- | --- | --- | --- | --- | --- | --- | --- | --- | --- | --- | --- | --- | --- | --- | --- | --- | --- | --- | --- | --- | --- | --- | --- | --- | --- | --- | --- | --- | --- | --- | --- | --- | --- | --- | --- | --- | --- | --- | --- | --- | --- | --- | --- | --- | --- | --- | --- | --- | --- | --- | --- | --- | --- | --- | --- | --- | --- | --- | --- | --- | --- | --- | --- | --- | --- | --- | --- | --- | --- | --- | --- | --- | --- | --- | --- | --- | --- | --- | --- | --- | --- | --- | --- | --- | --- | --- | --- | --- | --- | --- | --- | --- | --- | --- | --- | --- | --- | --- | --- | --- |

**Supplementary Table 2 Statistical table of sequencing volume per fungal sample**

| Sample | Input | Filtered | Denoised | Merged | Non-chimeric | Non-singleton |
| --- | --- | --- | --- | --- | --- | --- |
| UN1 | 134940 | 87770 | 87571 | 87457 | 87452 | 87452 |
| UN2 | 142496 | 128582 | 127037 | 125547 | 124417 | 124417 |
| UN3 | 142959 | 102227 | 101716 | 101145 | 97815 | 97815 |
| UN4 | 147608 | 133877 | 132897 | 131696 | 128389 | 128387 |
| UN5 | 138572 | 122705 | 122497 | 122076 | 121260 | 121260 |
| UN6 | 140938 | 129847 | 129292 | 128257 | 122650 | 122650 |
| UN7 | 139392 | 121131 | 119510 | 118219 | 115847 | 115847 |
| UN8 | 136332 | 125277 | 124547 | 123475 | 122223 | 122223 |
| UN9 | 135273 | 120555 | 119094 | 117615 | 111342 | 111342 |
| CN1 | 144713 | 133911 | 133245 | 132230 | 123850 | 123850 |
| CN2 | 143355 | 132336 | 131992 | 131412 | 130250 | 130250 |
| CN3 | 135474 | 124035 | 123681 | 122609 | 117445 | 117445 |
| CN4 | 134660 | 123213 | 122172 | 121163 | 115592 | 115592 |
| CN5 | 139018 | 119950 | 119514 | 119091 | 115221 | 115221 |
| CN6 | 147436 | 136481 | 135895 | 135340 | 127496 | 127496 |
| CN7 | 138748 | 125413 | 124823 | 123213 | 112964 | 112964 |
| CN8 | 138042 | 127560 | 126991 | 126002 | 122233 | 122232 |
| CN9 | 139444 | 129656 | 129380 | 129108 | 128048 | 128048 |

## Supplementary Figures

| **(A)**  **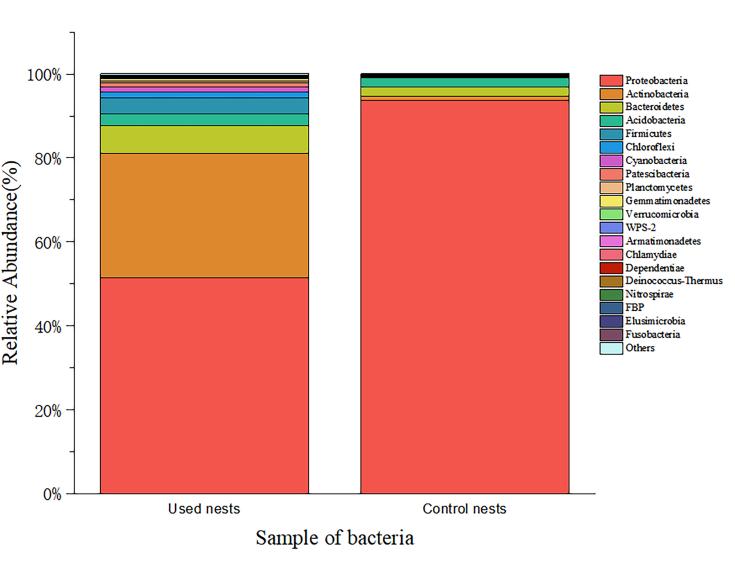** | **(B)**  **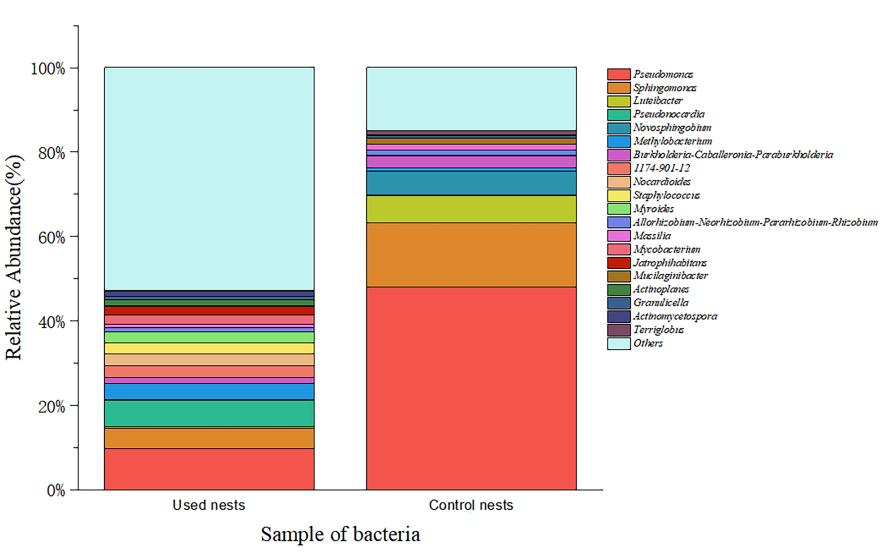** |
| --- | --- |

**Supplementary Figure 1.** The relative abundance of bacteria. **(A)** Bacteria composition in all samples at the phylum level. **(B)** Bacteria composition in all samples at the genus level.

| **(A)**  **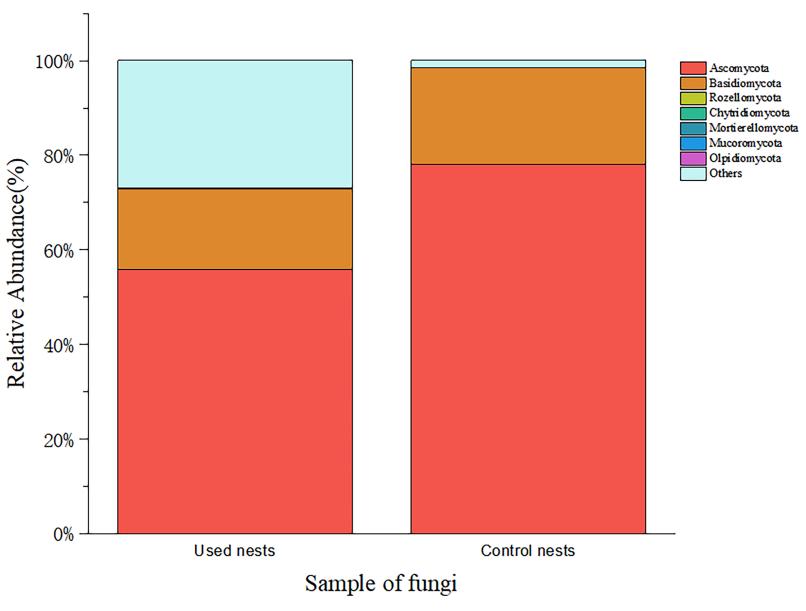** | **(B)**  **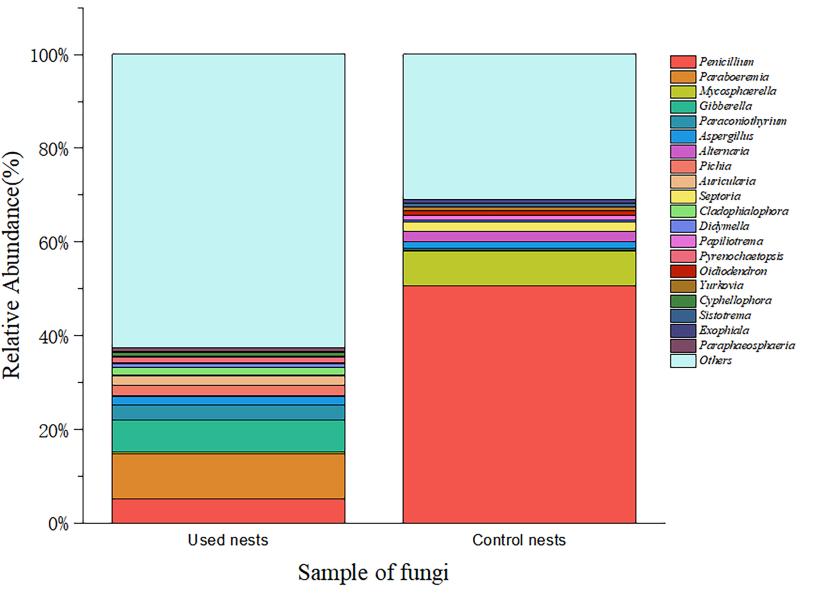** |
| --- | --- |

**Supplementary Figure 2.** The relative abundance of fungi. **(A)** Fungi composition in all samples at the phylum level. **(B)** Fungi composition in all samples at the genus level.


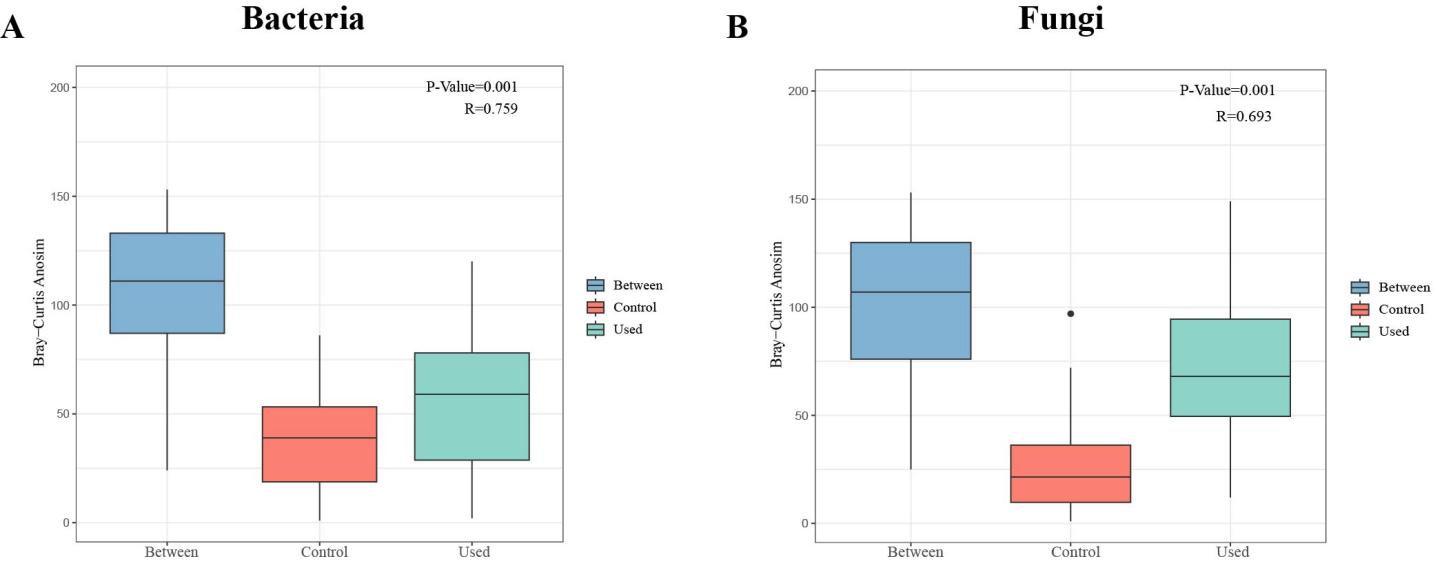


**Supplementary Figure 3.** ANOSIM analysis. The x-axis represents the results between and within groups, and the y-axis represents the distance rank between the samples. R-value: R>0 indicates that the inter-group difference is greater than intra-group, R<0 indicates that inter-group difference is less than intra-group. P-value: P< 0.05 reflects a statistically significant difference. (A) Bacteria. (B) Fungi.
